# An estimate of the pace of aging from a single brain scan predicts chronic disease, dementia, and mortality

**DOI:** 10.1093/geroni/igaf122.2268

**Published:** 2025-12-31

**Authors:** Ethan Whitman, Maxwell Elliott, Annchen Knodt, Terrie Moffitt, Avshalom Caspi, Ahmad Hariri

**Affiliations:** Duke University, Durham, North Carolina, United States; University of Minnesota, Minneapolis, Minnesota, United States; Duke University, Durham, North Carolina, United States; Duke University, Durham, North Carolina, United States; Duke University, Durham, North Carolina, United States; Duke University, Durham, North Carolina, United States

## Abstract

Neuroimaging is a common, non-invasive measure, making it a desirable target for developing aging biomarkers. Current MRI-based aging biomarkers (i.e., brain-age gap) are estimated from cross-sectional associations with chronological age - akin to “first-generation” epigenetic clocks. DunedinPACE, a “third-generation” epigenetic measure, has advantages over first-generation epigenetic clocks because it directly estimates longitudinal aging. We extend this approach to neuroimaging with a “next-generation” neuroimaging-based biomarker of the rate of biological aging. The pace of whole-body aging was determined by tracking physiological decline of 6 organ systems over 20 years in Dunedin Study members. An elastic-net regression model was trained to estimate the pace of whole-body aging using neuroimaging data collected at age 45 from 860 Study members. We call this measure the Dunedin Pace of Aging Calculated from NeuroImaging or “DunedinPACNI.” We exported DunedinPACNI to the UK Biobank and ADNI to test associations with disease and decline. DunedinPACNI estimated whole-body aging with a cross-validated accuracy of r = 0.42 in Dunedin Study members. In 1,737 ADNI participants, people with faster DunedinPACNI had greater cognitive impairment (MCI: β = 0.27; p < 0.001; AD: β = 0.81; p < 0.001) dementia risk (HR = 1.76, p < 0.001), and hippocampal atrophy (β=-0.15; p < 0.001). In 42,583 UK Biobank participants, people with faster DunedinPACNI also had greater hippocampal atrophy (β=-0.09; p < 0.001), frailty (β = 0.17; p < 0.001), disease risk (HR = 1.14, p = 0.01), and mortality risk (HR = 1.32, p < 0.001). DunedinPACNI was superior or similar to brain-age gap at predicting clinical outcomes. DunedinPACNI, a novel, exportable MRI biomarker for longitudinal whole-body aging, offers improved opportunity to measure aging from brain structure.

